# An Empirical Study of Informed Refusal by Sexual Assault Survivors Under the Protection of Children from Sexual Offences (POCSO) Act at a Tertiary Care Center in Northeast India

**DOI:** 10.7759/cureus.86709

**Published:** 2025-06-25

**Authors:** Oli Goswami, Putul Mahanta, Narayan C Sharma, Bharati Basumatari, Rajib Ray Baruah, Alaka Das

**Affiliations:** 1 Forensic Medicine, Employees' State Insurance Corporation (ESIC) Medical College, Guwahati, Guwahati, IND; 2 Forensic Medicine and Toxicology, Nalbari Medical College (NMC), Nalbari, IND; 3 Pediatrics, PA Sangma International Medical College and Hospital, Khanapara, IND; 4 Radiology, Nalbari Medical College and Hospital (NMCH), Nalbari, IND; 5 Pediatric Surgery, Gauhati Medical College & Hospital, Guwahati, IND; 6 Biochemistry, Assam Medical College, Dibrugarh, IND

**Keywords:** demographic profile, medical examination, proper counseling, rape survivors, reason for refusal

## Abstract

Introduction

In India, the Protection of Children from Sexual Offences (POCSO) Act is a comprehensive legislative framework designed to protect children from sexual abuse, emphasizing child-friendly processes and mandatory reporting to ensure justice and protection. This study aims to evaluate the prevalence, demographic patterns, and various factors that lead to the refusal of medical examinations by sexual assault survivors despite appropriate counseling.

Methods

This hospital-based cross-sectional study was conducted at Gauhati Medical College and Hospital (GMCH) in Guwahati, Assam, from January 6, 2019, to January 6, 2021. The study included all sexual assault survivors under the POCSO Act who refused medical examination at the Department of Forensic Medicine. Survivors brought under the POCSO Act who consented to the examination were excluded.

Results

A total of 350 survivors under the POCSO Act were presented to the examination center for examination by the law enforcement agency (police), of which only two were male survivors. One hundred and two cases refused examination, representing 29.14% of the total cases. Of these 102 cases, 67 (65.7%) survivors declined examination. At the same time, parents of minors refused on behalf of their wards in 35 (34.3%) cases. Romantic involvements (53.7%, n=36), involvement in child marriage (19.4%, n=13), social stigma (4.5%, n=3), and fear of examination procedure (14.9%, n=10) were the most reported causes of refusal by the survivors. The age of the survivor and the reason for refusal were found to be associated (chi-square value: 10.3, p-value < 0.05). Meanwhile, parents or guardians mostly refused examination due to fear of the procedure (45.7%, n = 16) and social stigma (31.4%, n = 11). Parents' reasons for refusal were found to be significantly associated with the age (chi-square value: 23.7, p-value < 0.001) and education level (chi-square value: 15.0, p-value = 0.005) of the survivor.

Conclusion

Romantic relationships with the alleged assaulter, child marriage, and fear of medical procedures were the noted reasons for refusal among the survivors. Parents of toddlers mostly refused a medical examination of their child, fearing the possible physical or psychological side effects of the procedure. Social stigma and fear also contributed to parental refusal. The reasons for refusal significantly differed with the age of the survivors. Identification and assessment of reasons for informed refusals to medical examination among sexual assault survivors is critical in determining the underlying factors of such refusals. Proper counseling may reduce refusal rates due to fear of the procedure.

## Introduction

Child sexual abuse is a menace that affects a child in all dimensions, be it physically, emotionally, or behaviorally. As per the existing laws in India, a sexual assault survivor under the age of 18 is provided a legal remedy under the Protection of Children from Sexual Offences (POCSO) Act. This act is gender-neutral legislation, and many of its provisions are survivor-friendly. Section 27 of the Act is related to the medical examination of the survivor. The survivor is examined in a child-friendly environment by a female doctor only. The examination is to be done within 24 hours, preferably as early as possible [1].

As per a recent report, more than 30% of females and 13% of males below 18 years were the victims of sexual violence in 2023 in India [2]. Sexual abuse has a lasting impact on a person's physical and mental health. In many cases, the victim, parent, or guardian may be afraid or stigmatized and choose not to file a complaint against the accused, but they still require medical-legal assessment and hospital care. Not only as advocates of child health, but medical practitioners also play an essential role in detecting child sexual abuse. However, the victim cannot be forced to undergo a medico-legal examination by the police or the court without the informed consent of the child, parent, or guardian [3].

Patient autonomy is a prerequisite to the ethical practice of medicine [4]. In the examination of children of sexual abuse, too, this principle is followed to the core. The right to consent to a medical examination lies with the children if they are above 12 years of age and are of sound mind. On the other hand, it lies with the parents or guardians to determine if the individual is below 12 years of age or of unsound mind, even after reaching the age of 12. The survivor, parents, or guardians may agree to or refuse a medical examination. As per Section 28 of Bharatiya Nyaya Sanhita (BNS), a child above 12 can provide legally valid consent or refusal for examination. No amount of coercion or any other unfair means can be used to obtain consent [5]. The right to refusal is an integral and important part of medical practice. The survivors, patients, or guardians may refuse the examination, provided they are fully aware of the nature of the examination and the consequences of such refusal.

The present study aims to find the prevalence, distribution, and factors behind the refusal of medico-legal examination by the survivors of sexual assault brought for medico-legal examination who voluntarily refused a medical examination in the Department of Forensic Medicine in Gauhati Medical College & Hospital (GMCH), Guwahati, Assam.

## Materials and methods

The hospital-based cross-sectional study was conducted among sexual assault survivors under the POCSO Act who were brought for a medico-legal examination in the Department of Forensic Medicine at GMCH, Guwahati, Assam, from January 6, 2019, to January 6, 2021. The study included all sexual assault survivors under the POCSO Act who refused medical examination to study various factors that influence informed refusal, including patient autonomy, communication between healthcare providers and patients, and the ethical considerations involving informed refusal. The study was conducted after obtaining approval from the institutional ethics committee, vide reference number MC/190/2007/Pt-11/Dec-18/24, dated January 5, 2019. Informed consent was obtained from parents or guardians in cases where the survivors were in the 0-12 age group or those above 12 with impaired mental capacity. At the same time, the survivors in the 12-18 age group gave their consent.

Inclusion criteria

All survivors under the POCSO Act who refused examination at the examination center.

Exclusion criteria

All survivors under the POCSO Act who gave consent for examination at the examination center.

The nature, purpose, and scope of the examination, including forensic evidence collection, medical evaluation, potential treatment options, and findings in legal proceedings, have been thoroughly explained to ensure the patient understands the process. The refusal is made voluntarily and without coercion, and the patient understands the potential legal implications of his decision. The study acknowledges that the patient has been offered a forensic medical examination following a reported or suspected sexual assault and seeks to find a remedy for the same. Strict confidentiality and existing protocols as per the POCSO Act were followed to ensure the safety and privacy of the participants.

The survivors or their parents/guardians were counseled about the details regarding the nature and purpose of the examination as per the guidelines and protocols of medico-legal care for survivors/victims of sexual violence set by the Ministry of Health and Family Welfare, Government of India. They were informed about the purpose of the medico-legal examination, which is to support the investigation, apprehension, and prosecution of the sexual offender. They were also told about the examination procedure involving an examination of the mouth, breasts, vagina, anus, and rectum as necessary, depending on the particular circumstances. It was also informed that collection of forensic evidence would be done with the consent of the survivor to aid in the investigation, which might require removing and isolating clothing, scalp hair, foreign substances from the body, saliva, pubic hair, samples taken from the vagina, anus, rectum, and mouth, and collecting a blood sample. It was also clearly stated that the survivor or their parent/guardian has the right to consent to or refuse the medical examination, evidence collection, or both, and that refusal will not result in denial of medical treatment to the survivor.

Data was documented in a pre-formatted proforma. The first part of the proforma included data on sociodemographic variables, including age, sex, place of origin, and education level of the survivor. The second part included the type of refusal and the reasons for refusal. The reasons for refusal by the survivors or their parents/guardians were documented after all aspects, whether legal or related to the examination process, were explained to them. Open-ended questions were included for documenting the reasons for refusals to elicit detailed responses.

Statistical analysis

The data were entered into Microsoft Excel (Microsoft Corporation, Redmond, WA, US) and analyzed using the Statistical Package for the Social Sciences (SPSS) version 21 (IBM Corp., Armonk, NY, US). The data distribution was represented in terms of frequencies and percentages. The association between the reasons for the refusal of medical examination and different sociodemographic variables was assessed using the chi-square test, considering a p-value below 0.05 as statistically significant.

## Results

A total of 350 sexual assault survivors under the POCSO Act were brought for a medico-legal examination to the department of forensic medicine of the tertiary care hospital during the 2-year study period, out of which only 2 were male survivors.

A total of 102 cases were refused examination, which accounted for 29.14% of the total cases. Of the 102 cases, 67 (65.7%) survivors themselves refused the examination. In the remaining cases (n = 35, 34.3%), the guardian or parents refused the examination. Among these 35 cases where consent was obtained from parents/guardians, 2 were mentally challenged and above the age of 12. The majority (99.0%; n = 101) of survivors who refused examinations were females. More than half of the survivors (n=69; 67.6%) belonged to the 12-to-18-year age group. The majority (64.7%; n = 66) of the survivors came from rural backgrounds. Almost 84% (n=86) of the survivors were literate, among whom the majority (32.4%; n=33) had completed education up to the secondary level (Table 1).

**Table 1:** Profile of the sexual assault survivors refusing examination The data has been represented as frequency (n) and percentage (%); total sample N=102

Variables	Categories	Frequency (n=102)	Percentage
Type of refusal	Survivor's refusal	67	65.7%
	Parent/guardian's refusal	35	34.3%
Age-group	0-6 years	15	14.7%
	6-12 years	18	17.6%
	12-18 years	69	67.6%
Gender	Male	01	1.0%
	Female	101	99.0%
Place of origin	Urban	36	35.3%
	Rural	66	64.7%
Educational qualifications	Yet to begin	5	4.9%
	Illiterate	11	10.8%
	Elementary	12	11.8%
	Lower primary	19	18.6%
	Upper primary	22	21.6%
	Secondary	33	32.4%

In 67 of the 102 instances, the survivors refused to undergo medical examination. Of the cases, a majority (36; 53.7%) denied examination due to a romantic relationship with the offender. Ten (14.9%) survivors cited fear of the examination procedure as their reason for refusal. The sense of shame accounted for refusal in 5 (7.5%) cases. A total of 13 (19.4%) of the survivors who declined exams were involved in child marriages. Another 3 (4.5%) cases refused examination, fearing social stigma (Table 2).

**Table 2:** Reasons for survivor's refusal The data are represented as frequency (n) and percentage (%), with a total sample size of N = 67.

Reasons for the survivor's refusal	Total no (N=67)	Percentage
Romantic	36	53.7%
Shamefulness	05	07.5%
Fear of medical examination	10	14.9%
Child marriage	13	19.4%
Social stigma	03	04.5%

The guardians or parents refused examination in 35 (34.3%) cases. The majority (16 cases; 45.7%) of parents/guardians refused because they were frightened by the examination process. Social stigma was the next important factor in this group, which comprised 11 cases (31.4%). The third reason for refusal was the parents' fear that their wards might be expelled from their schools. It constituted 6 (17.14%) cases. Two (5.72%) of the children were mentally challenged; their refusal was given by their parents (Table 3).

**Table 3:** Reasons for refusal by parents/guardians The data are represented as frequency (n) and percentage (%); total sample size, N = 35.

Reasons for the parent/guardian's refusal	Total No. N = 35	Percentage
Fear of medical examination	16	45.7
Social stigma	11	31.4
Fear of expulsion from school	06	17.1
Mentally challenged	02	5.7

Of the 67 survivors who refused medical examination, the majority, 55 (82.1%), belonged to the 12- to 16-year-old age group. Romantic association with the offender (n=27, 49.1%), child marriage (n=13, 21.8%), and fear of medical procedure (n=10, 18.2%) were the most reported reasons for refusal in this age group. While the majority, 9 out of 12 survivors (75.0%), belonging to the 16-18 years age group, refused due to romantic associations. No participant in the 16-18 years age group refused examination due to fear of the procedure. A significant association was observed between the age of the survivor and the reason for refusal (p < 0.05). However, no statistically significant association was observed between the reason for refusal by the survivor and their place of origin or education level (Table 4).

**Table 4:** Factors associated with reasons for refusal by the survivors The data are represented as frequency (n) and percentage (%), with a total sample size of N = 67. *p < 0.05 was considered statistically significant for the chi-square test.

Variables	Reason for refusal by survivor					Chi-square	p-value
	Romantic (n=36)	Fear of medical exam (n=10)	Child marriage (n=13)	Social stigma (n=3)	Shame (n=5)		
Age group							
12-16 years (n=55)	27 (49.1%)	10 (18.2%)	12 (21.8%)	1 (1.8%)	5 (9.1%)	10.3	0.036*
16-18 years (n=12)	9 (75.0%)	0 (0.0%)	1 (8.3%)	2 (16.7%)	0 (0.0%)		
Place of origin							
Rural (n=49)	24 (49.0%)	9 (18.4%)	11 (22.4%)	2 (4.1%)	3 (6.1%)	3.6	0.46
Urban (n=18)	12 (66.7%)	1 (5.6%)	2 (11.1%)	1 (5.6%)	2 (11.1%)		
Education							
Illiterate (n=9)	4 (44.4%)	3 (33.3%)	1 (11.1%)	0 (0.0%)	1 (11.1%)	6.4	0.59
Up to primary (n=25)	11 (44.4%)	4 (16.0%)	7 (28.0%)	1 (4.0%)	2 (8.0%)		
Up to secondary (n=33)	21 (63.6%)	3 (9.1%)	5 (15.2%)	2 (6.1%)	2 (6.1%)		

In cases where parents/guardians of the survivors refused medical examination, except for the two mentally challenged cases, reasons for refusal were found to be significantly associated with the age of the survivor (p-value < 0.001) and education (p-value = 0.005). Parents of all the survivors below 4 years of age refused due to fear of the procedure (n = 8, 100.0%). In cases where the survivors were between the ages of 4 and 8, parents largely declined because of societal stigma (n = 8, 57.1%). Parental refusal was mostly noted due to fear of expulsion from school among survivors who were 8 to 12 years of age (n = 6, 54.5%). In the case of the survivors who were yet to begin their education (n = 5, 100.0%) or at the elementary level of schooling (n = 8, 66.7%), parents mostly refused medical examination due to fear of the procedure. Parental refusal due to social stigma (n = 7, 43.8%) and fear of expulsion from school (n = 6, 37.5%) were most commonly noted among those studying at the lower primary level (Table 5).

**Table 5:** Factors associated with reasons for refusal by the parents/guardians The data are represented as frequency (n) and percentage (%), with a total sample size of N = 33. Two mentally challenged cases were not included in the analysis. *p < 0.05 was considered statistically significant for the chi-square test.

Variables	Reason of refusal by parents/guardians			Chi-square value	p-value
	Fear of medical exam (n=16)	Social stigma (n=11)	Fear of expulsion from school (n=6)		
Age group					
0-4 years (n=8)	8 (100.0%)	0 (0.0%)	0 (0.0%)	23.7	<0.001*
4-8 years (n=14)	6 (42.9%)	8 (57.1%)	0 (0.0%)		
8-12 years (n=11)	2 (18.2%)	3 (27.3%)	6 (54.5%)		
Place of origin					
Rural (n=15)	8 (53.3%)	6 (40.0%)	1 (6.7%)	2.5	0.29
Urban (n=18)	8 (44.4%)	5 (27.8%)	5 (27.8%)		
Education					
Yet to begin (n=5)	5 (100.0%)	0 (0.0%)	0 (0.0%)	15.0	0.005*
Elementary (n=12)	8 (66.7%)	4 (33.3%)	0 (0.0%)		
Lower primary (n=16)	3 (18.8%)	7 (43.8%)	6 (37.5%)		

## Discussion

In response to the increasing prevalence of child sexual abuse in India, the Ministry of Women and Child Development introduced the POCSO Act 2012. The POCSO Act provides special courts for expedited trials, offers legal recourse for sexual abuse and exploitation, and integrates child-friendly practices. Given the POCSO Act of 2012, doctors have a dual job that includes both diagnosing child abuse and providing medical care [1]. Medical evidence is corroborative only. Sexual assault frequently has very few outward manifestations. However, for the protection of abused children, physical findings must be appropriately determined, documented, and interpreted in light of current scientific understanding [6,7]. The autonomy of child sexual assault survivors to refuse a medical examination needs to be respected. However, in the absence of a medical investigation report or in instances when the alleged sexual assault victim refuses to undergo a medical examination, it can lead to negative interpretations against the victim. It would offer the suspected rape accused the benefit of the doubt. As per a recent report published in the Hindu, the Honourable Madras High Court made it clear that a medical examination would be required only if there was penetrative sexual assault or if the victim had sustained injuries [8].

Frequently, children suffer in solitude and don't share their horrific experiences. Child sexual abuse survivors could think of themselves as broken, dissimilar, and unclean. For various emotional, social, and cultural reasons, they might struggle to express their feelings and experiences [9]. In a recent study, parents mentioned fear of being ostracised by society as a significant reason for not pursuing legal recourse. The study cited that the fear of social ostracism was the reason for the survivors' refusal of examination, at a rate of 3.25%. In contrast, in cases where parents or guardians gave consent, the fear of ostracism rose to 26.92% [10]. The findings are comparable to our study.

Parents' reasons for refusal were found to be significantly associated with the age of the survivor (p-value<0.001) and education (p-value = 0.005). Refusal due to fear of the procedure was mostly noted among parents of toddlers and preschoolers. They thought the process of examination may cause serious and permanent damage to the child's genitalia, which is already sore owing to the alleged sexual assault. In general, acute care, hospitalization, medical procedures, and preventive clinic visits are frequent instances of treatment that can be emotionally distressing and traumatic for pediatric patients [11]. The forensic medical and gynecological examination following sexual assault is a necessary practice, yet it may cause the victim further trauma [12]. However, when it comes to forensic DNA evidence, care must be taken to improve policies and procedures for accurate documentation, immediate medical assessment, and evidence gathering in cases of sexual assault involving prepubertal victims before medical evidence is harmed [13]. Also, parents of survivors above the age of four mostly refused medical examinations due to social stigma and fear of expulsion from school. Previous studies have suggested that perceived parental support acts as a protective factor among child sexual assault survivors [14]. To lessen the traumatic effects on the victimized child, parents who have children exposed to abuse must be empowered [15].

In instances where survivors refused medical examination, fear of the examination procedure was noted as the reason for refusal in 10 (14.9%) cases. Fear ranged from reclining on the examination bed in the lithotomy position to the collection procedure of the swab. The sense of shame that comes with exposing one's private parts to strangers, including medical professionals, was another significant factor that accounted for refusal in 5 (7.5%) cases. Another 3 (4.5%) cases refused examination, fearing social stigma. They did not want the matter to flare up. They also felt that if they regularly visited the courts, police stations, or hospitals for follow-up visits, then their neighbors might become aware of the matter. This may, in turn, harm their future marital prospects or marital life. It is documented that child sexual abuse victims who believed that care was unnecessary either downplayed their need for care or were unaware of the negative health effects of abuse. Repercussions were expected to include interruptions to family life, stigmatization, re-traumatizing medical treatment, privacy and control over disclosure, and retaliation from the offender [16]. A recent meta-analysis identified a strong correlation between sexual violence severity and trauma-related shame. Shame-addressing interventions might help survivors achieve better outcomes [17].

The age of the survivor and reason for refusal (p-value<0.05) were observed to be associated. Of the 67 survivors who refused medical examination, the majority, 55 (82.1%), belonged to the 12 to 16-year-old age group. Romantic involvement with the offender was the most reported reason for the survivor's refusal. These were the purported elopement cases in which the survivors were determined to protect their lovers from any legal ramifications. Consensual sexual relations under the POCSO Act and their ramifications have been widely discussed in a previous study [18]. Risks of delinquency were linked to romantic involvement during youth. Numerous research studies have demonstrated that adolescent dating violence is linked to health issues such as depression, anxiety, substance abuse, poor academic performance, and sexually transmitted infections (STIs) (STIs)/acquired immune deficiency syndrome (AIDS) [19]. Higher levels of controlling conduct in the relationship were connected with higher incidences of sexual and/or physical adolescent relationship abuse and similar acts of perpetration [20].

Almost 54% of the survivors in the current study were literate up to the upper primary or secondary level, indicating they were fully aware of their circumstances and the ramifications of their informed refusal to undertake any medical inquiry. Also, no participant in the 16-18 years age group refused examination due to fear of the procedure. The above observations might indicate that, in most cases, the sexual act happened with mutual consent. Batra N emphasized the necessity to respect the choices of those in the 16-18 age group in a broader and meaningful manner [21].

Even though marriage before 18 years of age is legally prohibited in India, one in every five girls and almost one in every six boys continue to get married before they attain the legal marriage age in India [22]. Thirteen (18.84%) of the survivors who declined exams were involved in child marriages. However, all these survivors, mostly aged 12-16 years, revealed their happiness with their marital life. They were defensive about their in-laws and husbands, wanting to save them from the law's clutches. A few of them also had children and feared that their children's lives would be at stake if they went for a medical examination. They also revealed that their advisors (both social and legal) have advised them against medical examinations. Girls who are in a child marriage but have not yet reached the legal marriageable age frequently engage in sexual encounters with their partner as a societal duty. Age, education, spouse's employment status, affluence, husband's alcohol usage, women's autonomy, decision-making, and freedom of movement are linked to sexual violence and control over one's sexuality [23]. A recent review on marital rape among Indian women documented that intimate partner sexual coercion was very common, ranging from 9% to 80%, while marital rape was between 2% and 56% [24]. Only 10% of victims are thought to report spousal sexual abuse, even though the majority of sexual violence in India takes place in marriages [25].

The medical examination of the sole male survivor, whom the investigating agency brought, was refused by his parents. He was seven years of age. The parents considered the matter trivial and could not understand the reason for the legal formalities. They were also ashamed that their son, being a male, was made a "survivor" in the case. The lower number of male survivors in most studies is a concerning trend. The few cases that are brought for examination reflect only the tip of the iceberg of the sexual abuse happening to male children. Indeed, data from the National Intimate Partner and Sexual Violence Survey, US, suggests that nearly one in four men in the US experienced some form of contact sexual violence in their lifetime [26]. Hegemonic masculinity is what most people would describe as 'being a man'. This attitude often prevents the reporting of sexual assault cases of males [27]. Patriarchy oppresses male children in Indian society, even preventing them from getting mental health treatment in collaborative child response units [28].

Medical evidence is immensely useful in cases of sexual assault when the victim is alive and even more so when the victim is dead. In many cases, the victim's assent to a medical examination is critical in determining the case's outcome. The legal structure ensures that investigations are conducted sensitively and effectively while preserving critical information for prosecution. Sensitivity needs to be preserved, especially in romantic situations. Survivors must be informed of their rights and legal obligations regarding medical examinations, as well as the importance of maintaining the integrity of the judicial process. The phenomenon of informed refusal during medical examinations under the POCSO Act highlights significant gaps in communication, consent, and trust. Establishing survivor-centered approaches that prioritize autonomy while ensuring access to justice is crucial. Strengthening legislative frameworks, training healthcare practitioners in trauma-informed care, and increasing awareness among stakeholders can help reduce refusal rates and improve outcomes for survivors. Furthermore, collaboration among legal, medical, and social sectors will be essential in fostering a supportive environment that respects survivors' rights and aids in their healing process.

Limitation

The present study findings highlight the prevalence of informed refusal for medical examination among sexual assault survivors under the POCSO Act and the underlying reasons for refusal by the survivors or their parents/guardians. Medical examination and counseling may play a useful role in ensuring the survivors' physical well-being and determining the outcome of the legal proceedings. The present findings provide important takeaways from the scarce literature regarding the reasons for refusal of medical examination among sexual assault survivors. However, the single-center design of the study limits the broader applicability of the findings. Multi-center studies with a qualitative approach may help identify the experiences of survivors that affect their refusal of medical examination. Also, the limited male representation in the current study might be a result of low reporting of such incidents against male children. Additionally, the study primarily focused on understanding the burden and associated factors of medical refusal, but it does not assess the quality of counseling provided, which is critical given its influence on refusal. More elaborate studies with a multicentric approach might be beneficial in understanding the true prevalence of informed medical examination refusal and its underlying factors among sexual assault survivors in the region.

## Conclusions

Refusal for medical examination by survivors owing to romantic contact with the perpetrator, particularly if the victim is literate, may indicate a consensual sexual relationship. Despite legal prohibition, the high prevalence of child marriage among survivors is concerning.

Appropriate counseling could aid in lowering the percentage of refusals brought on by procedure anxiety. Finding the underlying causes of sexual assault survivors' informed refusals to undergo medical examinations requires identifying and evaluating the reasons behind these refusals. It is important to promote early sex education, conduct awareness-raising efforts about the dangers of unprotected sex, and provide information about the legal options available to victims of sexual assault. The effectiveness of the medical examiner's exercise and responsibilities may be increased by a thorough assessment of the reasons why survivors of sexual assault may voluntarily refuse medical examinations.
